# Supplementary material for: Predicting MEG resting-state functional connectivity from microstructural information
Source: Netw Neurosci. 2021 Jun 3;5(2):477–504. doi: 10.1162/netn_a_00187 (PMC8233113; doi:10.1162/netn_a_00187)
Supplement: Supplementary file 1 [file netn-05-477-s001.pdf]

Messaritaki, E., Foley, S., Schiavi, S., Magazzini, L., Routley, B., Jones, D. K. & Signh, K. D. (2021). Supporting information for "Predicting MEG resting-state functional connectivity from microstructural information." *Network Neuroscience.* Advance publication. https://doi.org/10.1162/netn_a_00187

Supporting Information for **"Predicting MEG resting-state functional connectivity using microstructural information"**

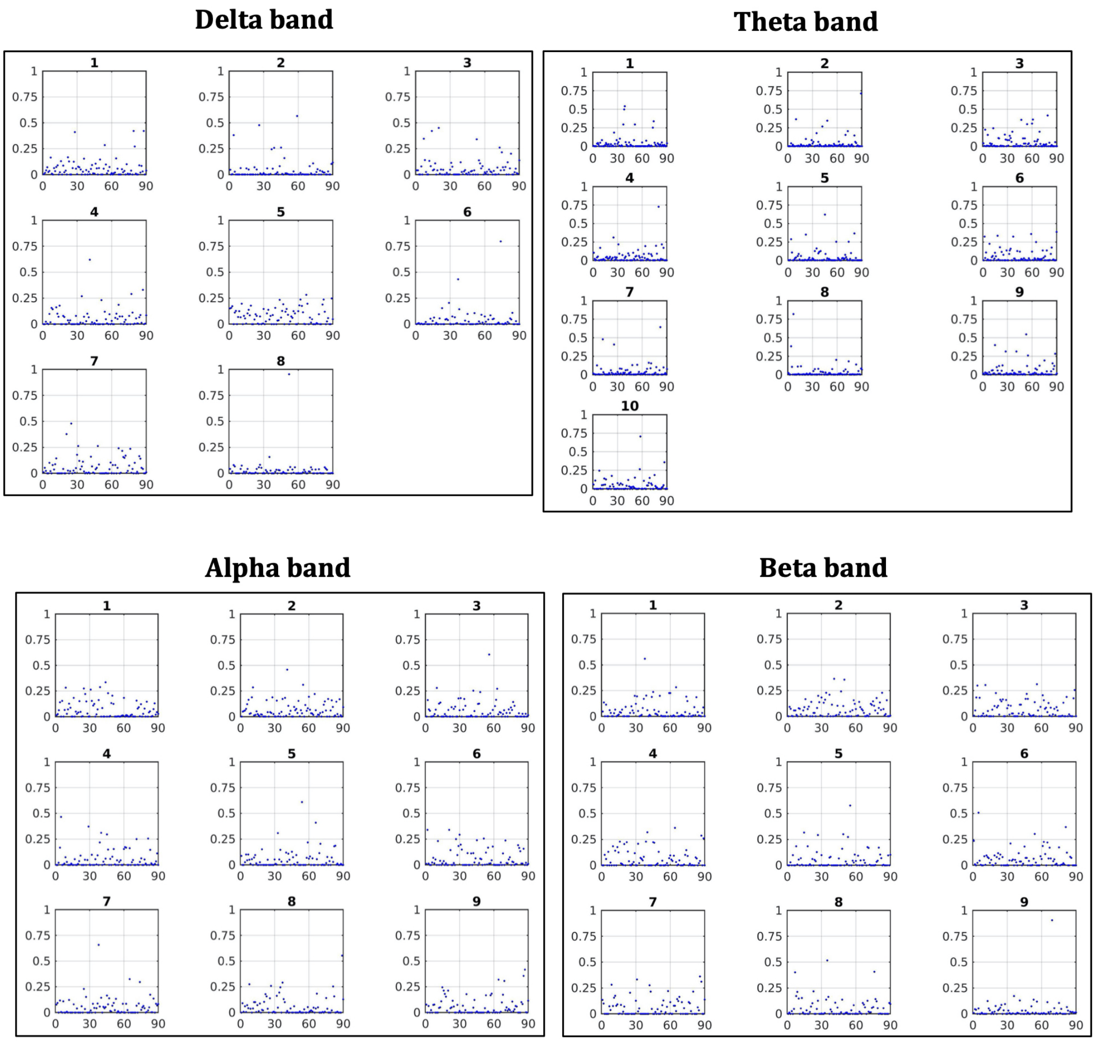

Figure 1: Relative contribution from each participant to each component of the FC_o, for the four frequency bands. The horizontal axis is over the 90 participants.

1	Precentral L	19	Supp Motor Area L	37	Hippocampus L	55	Fusiform L	73	Putamen L
2	Precentral R	20	Supp Motor Area R	38	Hippocampus R	56	Fusiform R	74	Putamen R
3	Frontal Sup L	21	Olfactory L	39	ParaHippocampal L	57	Postcentral L	75	Pallidum L
4	Frontal Sup R	22	Olfactory R	40	ParaHippocampal R	58	Postcentral R	76	Pallidum R
5	Frontal Sup Orb L	23	Frontal Sup Medial L	41	Amygdala L	59	Parietal Sup L	77	Thalamus L
6	Frontal Sup Orb R	24	Frontal Sup Medial R	42	Amygdala R	60	Parietal Sup R	78	Thalamus R
7	Frontal Mid L	25	Frontal Med Orb L	43	Calcarine L	61	Parietal Inf L	79	Heschl L
8	Frontal Mid R	26	Frontal Med Orb R	44	Calcarine R	62	Parietal Inf R	80	Heschl R
9	Frontal Mid Orb L	27	Rectus L	45	Cuneus L	63	SupraMarginal L	81	Temporal Sup L
10	Frontal Mid Orb R	28	Rectus R	46	Cuneus R	64	SupraMarginal R	82	Temporal Sup R
11	Frontal Inf Oper L	29	Insula L	47	Lingual L	65	Angular L	83	Temporal Pole Sup L
12	Frontal Inf Oper R	30	Insula R	48	Lingual R	66	Angular R	84	Temporal Pole Sup R
13	Frontal Inf Tri L	31	Cingulum Ant L	49	Occipital Sup L	67	Precuneus L	85	Temporal Mid L
14	Frontal Inf Tri R	32	Cingulum Ant R	50	Occipital Sup R	68	Precuneus R	86	Temporal Mid R
15	Frontal Inf Orb L	33	Cingulum Mid L	51	Occipital Mid L	69	Paracentral Lobule L	87	Temporal Pole Mid L
16	Frontal Inf Orb R	34	Cingulum Mid R	52	Occipital Mid R	70	Paracentral Lobule R	88	Temporal Pole Mid R
17	Rolandic Oper L	35	Cingulum Post L	53	Occipital Inf L	71	Caudate L	89	Temporal Inf L
18	Rolandic Oper R	36	Cingulum Post R	54	Occipital Inf R	72	Caudate R	90	Temporal Inf R

Table 1: AAL atlas areas.

freq. band	NS	ED	FA	ED	bin	ED	MM	ED	RD	ED
delta	-0.005	-0.840	-0.035	-0.894	-0.083	-0.821	-0.001	-0.920	-0.047	-0.800
theta	-0.006	-0.551	-0.046	-0.632	-0.097	-0.549	-0.002	-0.672	-0.052	-0.532
alpha	-0.015	-0.838	-0.069	-1.034	-0.197	-0.852	-0.005	-1.101	-0.011	-0.817
beta	-0.017	-0.963	-0.092	-1.175	-0.231	-0.969	-0.006	-1.259	-0.118	-0.943

Table 2: Mean (over the 90 participants) value of the coefficients that result from the SPL algorithm, when each of the structural metric is used as a predictor with the Euclidean distance, for each frequency band.

freq. band	NS	ED	FA	ED	bin	ED	MM	tED	RD	ED
delta	-0.006	-0.790	-0.005	-0.872	-0.009	-0.811	-0.004	-0.874	-0.009	-0.801
theta	-0.006	-0.523	-0.005	-0.619	-0.10	-0.541	-0.006	-0.592	-0.010	-0.535
alpha	-0.013	-0.798	-0.005	-1.031	-0.019	-0.848	-0.013	-0.935	-0.019	-0.837
beta	-0.015	-0.903	-0.012	-1.132	-0.023	-0.950	-0.016	-1.048	-0.023	-0.939

Table 3: Mean (over the 90 participants) value of the coefficients that result from the SI algorithm, when each of the structural metric is used as a predictor with the Euclidean distance, for each frequency band.

Components	SC edge weightings									
	NS-FA	NS-bin	NS-MM	NS-RD	FA-bin	FA-MM	FA-RD	bin-MM	bin-RD	MM-RD
1	4e-04	8e-08	1e-04	2e-10	2e-18	**0.253**	4e-16	7e-15	4e-4	2e-15
2	2e-08	4e-08	3e-06	1e-07	**0.348**	2e-12	**0.247**	1e-12	8e-03	8e-12
3	**0.344**	1e-09	0.014	1e-08	2e-14	7e-03	5e-12	5e-10	**0.851**	2e-09
4	9e-11	2e-03	**0.082**	0.018	3e-09	4e-06	3e-08	**0.368**	**0.280**	**0.695**
5	5e-07	0.013	2e-08	1e-03	5e-15	**0.281**	2e-14	3e-12	**0.058**	8e-13
6	4e-11	8e-05	5e-07	7e-06	3e-14	1e-10	6e-09	**0.196**	9e-03	**0.450**
7	6e-07	6e-13	1e-04	5e-14	3e-17	4e-05	2e-17	2e-15	2e-04	7e-16
8	7e-04	0.017	0.015	1e-03	3e-07	0.018	3e-07	3e-05	0.021	8e-06
FC_o	**0.059**	**0.182**	**0.866**	**0.100**	**0.339**	**0.063**	**0.748**	**0.241**	**0.266**	**0.152**

Table 4: *p*-values resulting from the comparison of the correlation distributions, for each pair of structural edge weightings for the SPL algorithm, in the delta band. Bold numbers indicate the cases for which the distributions were *not* statistically significantly different.

Components	SC edge weightings									
	NS-FA	NS-bin	NS-MM	NS-RD	FA-bin	FA-MM	FA-RD	bin-MM	bin-RD	MM-RD
1	8e-14	1e-10	6e-08	2e-10	5e-15	5e-14	8e-10	7e-07	**0.286**	2e-06
2	9e-09	3e-08	2e-12	4e-07	0.013	3e-15	**0.162**	4e-15	**0.704**	2e-14
3	2e-07	1e-04	1e-09	3e-05	8e-04	5e-03	**0.155**	2e-06	0.008	2e-03
4	1e-04	2e-09	**0.838**	6e-10	1e-06	3e-05	3e-06	5e-09	0.054	3e-09
5	8e-07	1e-05	5e-10	1e-07	8e-04	6e-14	**0.093**	2e-13	2e-05	1e-13
6	5e-14	9e-13	1e-05	1e-12	2e-11	2e-15	3e-04	1e-13	2e-03	1e-12
7	4e-07	2e-05	4e-15	1e-04	0.010	4e-17	**0.201**	2e-16	**0.860**	6e-15
8	5e-04	9e-04	1e-09	0.009	**0.184**	8e-12	**0.092**	4e-12	**0.278**	6e-11
9	**0.103**	5e-06	**0.188**	2e-04	3e-11	**0.878**	2e-09	1e-04	0.007	0.001
10	**0.283**	0.001	**0.046**	0.003	3e-07	**0.372**	3e-06	2e-04	**0.679**	4e-04
FC_o	**0.066**	**0.053**	**0.763**	**0.022**	**0.973**	**0.037**	**0.664**	**0.085**	**0.457**	**0.044**

Table 5: *p*-values resulting from the comparison of the correlation distributions, for each pair of structural edge weightings for the SPL algorithm, in the theta band. Bold numbers indicate the cases for which the distributions were *not* statistically significantly different.

Components	SC edge weightings									
	NS-FA	NS-bin	NS-MM	NS-RD	FA-bin	FA-MM	FA-RD	bin-MM	bin-RD	MM-RD
1	2e-19	6e-16	1e-08	6e-16	2e-16	7e-19	2e-05	4e-13	2e-04	2e-13
2	1e-23	2e-15	3e-22	1e-13	8e-26	1e-16	4e-20	11e-09	**0.654**	1e-07
3	2e-09	4e-12	3e-12	3e-11	4e-07	2e-15	3e-05	2e-16	0.037	6e-16
4	3e-10	4e-09	2e-04	9e-09	2e-04	1e-15	**0.073**	3e-15	**0.719**	2e-14
5	4e-07	0.002	2e-11	0.010	4e-05	1e-12	8e-04	2e-11	**0.571**	3e-10
6	9e-09	0.049	0.021	5e-05	6e-15	5e-14	4e-05	1e-06	2e-07	2e-10
7	8e-12	9e-09	0.041	9e-07	7e-13	3e-15	5e-09	1e-10	**0.150**	2e-07
8	5e-10	2e-08	3e-08	2e-08	9e-07	3e-16	0.044	6e-16	**0.117**	1e-15
9	1e-09	0.022	**0.338**	**0.193**	6e-17	3e-11	9e-14	5e-04	0.024	0.036
FC_o	3e-05	2e-06	**0.689**	9e-08	**0.847**	6e-10	**0.410**	1e-07	**0.214**	1e-08

Table 6: *p*-values resulting from the comparison of the correlation distributions, for each pair of structural edge weightings for the SPL algorithm, in the alpha band. Bold numbers indicate the cases for which the distributions were *not* statistically significantly different.

Components	SC edge weightings									
	NS-FA	NS-bin	NS-MM	NS-RD	FA-bin	FA-MM	FA-RD	bin-MM	bin-RD	MM-RD
1	2e-16	6e-15	4e-03	1e-14	5e-12	2e-19	4e-05	6e-18	**0.277**	4e-16
2	2e-23	7e-15	2e-23	5e-14	1e-25	2e-12	3e-20	8e-13	**0.217**	2e-09
3	2e-10	6e-16	5e-15	2e-15	2e-14	7e-20	2e-11	1e-21	0.049	2e-21
4	3e-09	1e-06	1e-12	5e-08	3e-07	2e-17	**0.160**	4e-16	1e-03	7e-16
5	8e-10	6e-05	**0.077**	2e-08	3e-11	7e-12	0.027	2e-05	2e-09	3e-09
6	6e-04	7e-03	4e-11	4e-03	0.021	5e-12	**0.235**	3e-12	**0.543**	8e-12
7	1e-08	**0.681**	2e-07	4e-03	3e-14	2e-12	7e-10	2e-06	5e-09	1e-09
8	0.022	**0.900**	1e-11	1e-05	1e-04	6e-11	0.007	4e-10	1e-10	4e-13
9	1e-06	6e-04	**0.558**	7e-06	1e-04	7e-09	**0.303**	3e-05	2e-05	9e-07
FC_o	**0.079**	4e-04	**0.098**	1e-05	0.016	9e-05	0.002	7e-07	0.016	1e-08

Table 7: *p*-values resulting from the comparison of the correlation distributions, for each pair of structural edge weightings for the SPL algorithm, in the beta band. Bold numbers indicate the cases for which the distributions were *not* statistically significantly different.
